# Clusters of Hantavirus Infection, Southern Argentina

**DOI:** 10.3201/eid1301.060404

**Published:** 2007-01

**Authors:** Maria E. Lázaro, Gustavo E. Cantoni, Liliana M. Calanni, Amanda J. Resa, Eduardo R. Herrero, Marisa A. Iacono, Delia A. Enria, Stella M. González Cappa

**Affiliations:** * Hospital Zonal Bariloche, Bariloche, Argentina; † Unidad Regional de Epidemiología y Salud Ambiental, Bariloche, Argentina; ‡ Hospital Castro Rendón, Neuquén, Argentina; § Hospital de Área El Bolsón, El Bolsón, Argentina; ¶ Instituto Nacional de Enfermedades Virales Humanas, Pergamino, Argentina; # Universidad de Buenos Aires, Buenos Aires, Argentina

**Keywords:** Hantavirus, Andes virus, cluster, interhuman transmission, hantavirus pulmonary syndrome, transmission, epidemiology, outbreak, Argentina, acute respiratory distress syndrome, research

## Abstract

Person-to-person transmission of a hantavirus was first confirmed during a 1996 outbreak of hantavirus pulmonary syndrome in southern Argentina, where Andes virus is endemic. To identify other episodes of secondary transmission, we reviewed reports of 51 hantavirus infection cases from this region (November 1993–June 2005). Nine clusters involving 20 cases (39.2%) were found. Two patients, who had symptoms 3 weeks after they shared risks for rodent exposure, were considered a cluster. The other 8 clusters each began with an index case, which was almost always fatal, followed 19–40 days later by the illness of >1 person who had close and prolonged contact with the index case-patient. Person-to-person transmission was considered the probable source of these 8 clusters. The probability of initiating secondary cases was 41% for patients who died versus 4% for those who survived (p = 0.005). Interpersonal transmission of Andes virus infection should be considered even when rodent exposure cannot be definitively excluded.

## Abstract 2

Person-to-person transmission should be suspected when Andes virus case-patients are linked.

Hantaviruses (family *Bunyaviridae,* genus *Hantavirus*) are rodentborne, zoonotic, lipid-enveloped RNA viruses. Old World hantaviruses are associated with hemorrhagic fever with renal syndrome (HFRS), whereas New World hantaviruses cause hantavirus pulmonary syndrome (HPS) (*1*). Transmission to humans is thought to occur predominately by inhalation of infected rodent excreta (*2*). Person-to-person transmission was first documented in 1996, when an HPS outbreak due to Andes virus in southern Argentina provided reliable evidence for person-to-person transmission of a hantavirus (*3*–*5*). In this region, HPS was recognized in 1995 when an outbreak of respiratory illness in a family was investigated, and Andes virus was identified from autopsy tissues of one of the case-patients (*6*,*7*). Until now, Andes virus (reservoir *Oligoryzomys longicaudatus*) was the only hantavirus associated with human infections in this region and with most HPS cases reported in Chile (*8*,*9*).

The 1996 HPS outbreak in southern Argentina showed unique characteristics. The cases occurred in 3 cities over an 11-week period, and each case-patient had proven contact with another HPS case-patient. This unusual circumstance made it possible to identify the epidemiologic chain (*3*,*4*). An identical viral nucleotide sequence in HPS case-patients linked by interpersonal contact supported the hypothesis of person-to-person transmission (*5*). Serious outbreaks such as this are often fully investigated. However, investigation resources are usually limited for small outbreaks in HPS-endemic rural areas, so the epidemiologic diagnosis not easy to establish. To investigate the possibility of other episodes of person-to-person transmission, first proved in the 1996 outbreak, we reviewed the epidemiology of HPS and cluster formation in our region.

## Materials and Methods

**Figure 1:**
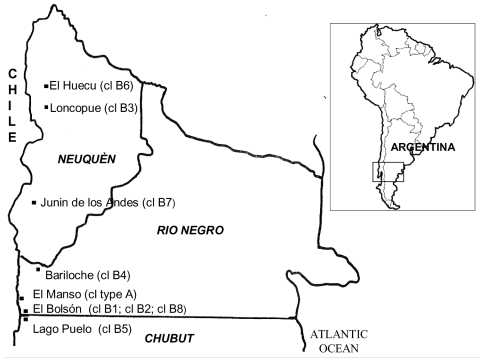
Geographic origin of clusters (cl) of Andes virus cases, southern Argentina.

The HPS-endemic southern area in Argentina is located in the western Patagonia region bordering Chile (Figure 1). The area consists of the western strip of Neuquen, Rio Negro, Chubut, and Santa Cruz provinces. Since 1995, information about all cases of hantavirus infection from Rio Negro and most of Neuquen has been collected by M.E. Lázaro. Systematic registry used standardized forms, including surveillance case reports, results of environmental and epidemiologic case investigations, and clinical data. This registry was used to identify clusters of hantavirus infections from November 1993 through June 2005. Case-patients who undoubtedly acquired the infection in the region (resided in the area >45 days before the onset of symptoms, had molecular evidence of Andes virus infection, or both) were considered in this review.

A cluster was defined as the association of a patient with confirmed HPS (index case-patient) with >1 contacts who showed laboratory evidence of hantavirus infection within 6 weeks of the onset of symptoms. Diagnostic confirmation was performed in referral centers (Instituto Nacional de Enfermedades Virales Humanas “Dr. Julio Maiztegui” and Instituto Nacional de Enfermedades Infecciosas “Dr. Carlos Malbran”). Serologic specimens were tested by ELISA for immunoglobulin M (IgM) and IgG antibodies. RNA was extracted from tissue, blood clots, or serum samples and amplified by reverse transcription (RT)–PCR. Viral genotype was characterized by sequencing the RT-PCR products.

When any HPS case was confirmed, an epidemiologic investigation of the places where the patient had lived, worked, or visited within the 6 weeks before the onset of symptoms was immediately conducted. To determine the most probable site of a patient’s exposure, we favored those where rodents were trapped, handled, or seen, or where rodent infestation was clearly evident (presence of excrements, nests, or gnawed food). As soon as a case was confirmed, rodent traps were set in the potential exposure sites. When a linked patient became ill, a new search that focused on identifying common or persistent rodent sources and possible interpersonal transmission was performed. A cluster that occurred in 1994 was studied retrospectively.

HPS case-patients who needed mechanical ventilation and hemodynamic support were considered to have a severe clinical form, while those that did not require such support were considered to have a moderate form. Infections without pulmonary involvement were considered mild forms.

Fisher exact test and Student *t* test were used to compare independent variables; p <0.05 was considered significant. Epidemiologic records of 43 case-patients from Rio Negro, 21 from Neuquen, and 3 from Chubut were reviewed.

Of the 67 total cases, 16 belonged to the 1996 outbreak and were excluded from this study. Of the 51 patients whose cases were reviewed, hantavirus infection was confirmed in 49. Acute infection was confirmed by detection of specific IgM antibodies in 47 patients. In 15 of them, the diagnosis was also confirmed by RT-PCR. Andes genotype was characterized for all 15 cases. For the other 2 cases, specific IgG confirmed past infection. The remaining 2 were potential HPS case-patients who died without confirmed diagnosis because of lack of samples but who were linked to close contacts with persons with confirmed infection.

Among the 51 cases, 9 clusters involving 20 patients (39.2%) were identified. Seven clusters met the strict cluster definition of confirmed acute infection because specific IgM was detected for 16 case-patients in these clusters. Each of the remaining 2 clusters was composed of a potential HPS case-patient (without confirmation due to lack of samples) who died and a household contact of that patient with confirmed infection.

## Results

Two types of cluster were observed and depended on the interval between cases (onset of symptoms). Type A consisted of infections with <1 week between cases; type B were those infections with >2 weeks between cases.

### Type A Clusters

Only 1 type A cluster was identified; it occurred in August 2002. It involved a 10-year-old male student from El Bolson and a 17-year-old male student from Bariloche. They became ill 21 and 23 days, respectively, after returning from a holiday week in El Manso, Rio Negro province, a rural area bordering Chile, where they had both participated in high-risk activities: hunting excursions, games in wilderness areas, and visits to stables. Both exhibited moderate forms of HPS and survived. No risks were identified in their respective houses in Bariloche and El Bolsón.

### Type B Clusters

**Figure 2:**
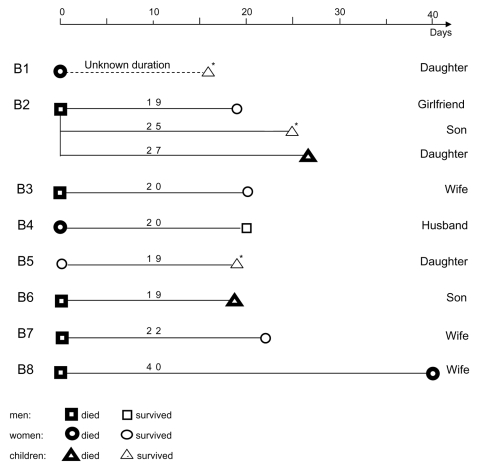
Type B clusters of Andes virus infection, southern Argentina.

Eight type B clusters, comprising 18 patients, were identified. Each was composed of an index case-patient, followed 19–40 days later by the disease in ≥1 household contact (Figure 2).

Cluster B1 took place in El Bolson, Rio Negro province, in September 1994. A 21-year-old housewife, who lived in a rural area and was breast-feeding her 7-month-old daughter, died after she experienced a flulike syndrome with acute respiratory distress. No samples were available for etiologic diagnosis. Eight months later, during a retrospective research study of contacts of persons with suspected HPS, the baby was studied because her mother fulfilled criteria for potential HPS. Antihantavirus IgG without specific IgM was detected in the baby, so a retrospective epidemiologic/ecologic study was performed. A second sample obtained 2 years and 9 months later confirmed past infection. Recollections by family members supported asymptomatic infection.

Cluster B2 occurred in El Bolson, Rio Negro province, in March and April 1995. A 38-year-old-man, who worked as soft drink distributor, died of HPS. Three members of his family showed laboratory evidence of acute infection with different degrees of clinical severity: 1) his 25-year-old pregnant girlfriend, a cashier in a supermarket, who did not live with him (moderate form, survived); 2) his 9-year-old son, a student, who lived with him (mild form, nonspecific fever syndrome); and 3) his 15-year-old daughter, a student, who lived with him (severe form, died). They became ill on the 19th, 25th, and 27th day respectively, after the onset of symptoms in the index case-patient. Some weeks before becoming ill, the man had gone on a fishing excursion with his girlfriend and son.

Cluster B3 took place in Loncopue, Neuquen province, in November and December 1997. A 41-year-old man, a rural worker with suspected HPS, died while being transferred to the hospital. Hantavirus infection was not confirmed because of lack of samples. He had captured and handled rodents and had observed rodent nests and excrement at home and work. He had also cleaned several unoccupied cabins. His wife, who was 31 years old, experienced symptoms 20 days later (severe form, survived).

Cluster B4 occurred in Bariloche, Rio Negro, in April and May 2000. A 26-year-old woman, who worked as gardener, died of HPS. She lived and worked in a rural area and had cleaned an uninhabited house 6 or 7 weeks before becoming ill. Her husband, a 63-year-old gardener, had onset of symptoms 20 days later (severe form, survived).

Cluster B5 happened in Lago Puelo, Chubut province, in May 2000. A 36-year-old woman was admitted to a hospital in Bariloche with a moderate form of HPS (survived). Her 3-year-old daughter, who was breast-feeding when her mother became ill, showed symptoms 19 days later (fever, vomiting, myalgia, and nasal congestion) but had no clinical or radiologic signs of pulmonary involvement (mild form). Seroconversion confirmed acute infection.

Cluster B6 occurred in Paraje El Morado, El Huecu, Neuquen province, in May and June 2000. A 46-year-old male farmer died of HPS. He was exposed to rodents at work and home. His 10-year-old son became ill 19 days later (severe form, died).

Cluster B7 occurred in Junin de los Andes, Neuquen province, in April and May 2001. A 42 year-old male veterinarian died of HPS. During the previous weeks he had been conducting ecologic research in wilderness areas in Neuquen and southern Chile. His wife, a 44-year-old teacher, became ill 20 days later (moderate form, survived). They were separated and lived in neighboring houses, but during the man’s illness he moved to his wife’s home where she took care of him.

Cluster B8 took place in El Bolson, Rio Negro province, in October and November 2003. A 31-year-old male construction worker died of HPS. During the previous weeks he had cleared weeds from a wilderness area and started building a cottage. His wife, a 28-year-old housewife, became ill 40 days later (severe form, died). She had not participated in the same potentially risky activities as her husband.

Type B clusters involved family groups. Clusters B2, B4, B5, B6, and B7 occurred during fall; clusters B1, B3, and B8 occurred in spring. All patients lived in rural or semirural areas. Each cluster was composed of 2 members, except B2, which comprised 4 (Figure 2). Ten (55.5%) patients were female. The average age was 28.3 ± 16.3 years (median 29.5 years; range 1–63 years); the baby of B1 was 1 year old when specific IgG was detected, but she had likely became infected at the age of 7 months, when her mother was sick with the disease. Children <16 years of age (28%) had secondary cases.

Andes genotype was identified in patients of cluster B2 (1 case), B3 (1 case), and B4 (2 cases). Index case-patients of clusters B3, B4, B6, B7, and B8 had occupational exposure. Exposure could have been either occupational or recreational for the index case-patient of B2. In B5, peridomestic and occupational exposure overlapped. In B1 (mother-baby), the study was retrospective without any information about rodent exposure.

Secondary cases occurred in household or intimate contacts of the respective index case-patients. Children had daily direct contact (e.g., kissing, touching, hugging, droplet spread) with their infected parents (B1, B2, B5, B6). Sexual intercourse was another route to be considered for persons in clusters B2, B3, B4, and B8. In B1 and B5, the mother was breast-feeding her child when she became ill. Airborne transmission cannot be excluded for any cluster.

When clusters B8 and B9 were studied, no evidence of rodents was found in or around the patients’ houses, and no small mammals were captured. All trapped rodents related to clusters B1 and B2 (49 rodents), B4 (9 rodents), and B5 (11 rodents) were seronegative for hantavirus. Information about captured rodents associated with clusters B3 and B6 was not available. In 6 of the 8 clusters, no evidence for rodent exposure by secondary case-patients was found. In the other 2 clusters (B3 and B6), rodent exposure was probable.

In 7 clusters, the average interval between symptom onset in index case-patients and in secondary case-patients was 23.4 ± 6.8 days (median 21 days; range 19–40 days) (Figure 2). In B1, diagnosis was retrospective, and infection was subclinical in 1 of the 2 members, so determining the interval was not possible.

Tested serum specimens from asymptomatic contacts of HPS index case-patients showed no evidence of hantavirus infection. The baby from cluster B1, who exhibited IgG antibodies, would be the exception. Contact surveillance identified 2 acute infections (mild forms) in children, by the presence of IgM antibodies (cluster B2) or by seroconversion (cluster B5).

All index case-patients had typical HPS symptoms. Among patients with secondary cases, 7 of 10 fulfilled the HPS definition. The remaining 3 were 2 children with mild forms (clusters B2 and B4) and a baby with asymptomatic infection (cluster B1). Index case-patients had a higher death rate than patients with secondary cases (87.5% vs 30%, p = 0.023). The death rate was also higher in index-case-patients than in patients not included in type B clusters (those with sporadic cases and cases in type A clusters) (87.5% vs 30%, p = 0.005) (Table).

**Table:** Case-fatality rate according to the type of cluster

Cluster	No. deaths/ no. cases	Case-fatality rate, %
Type B clusters		
All cases	10/18	55.6
Index cases	7/8	87.5
Secondary cases	3/10	30
Sporadic and type A cluster	10/33	30

Among the 51 patients whose cases were initially reviewed, 41 had undoubtedly acquired the infection from rodents (secondary case–patients excluded). Seventeen of these 41 died. Of the 17 patients who died, 7 (41%) had become an index case-patient of a cluster. In contrast, only 1 (4%) of the 24 patients who survived became an index case-patient (p = 0.005). These results indicate that the risk of initiating secondary cases was associated with the most severe manifestations.

Among the patients with sporadic cases and index patients who died, no differences in clinical characteristics were found. The mean number of days between onset of symptoms and death (6.78 ± 2.23 vs 6.86 ± 1.46, respectively; p = 0.9) was similar for both groups.

## Discussion

Both Old and New World hantavirus infections usually occur as sporadic cases (*10*). Although several persons frequently are exposed to the same risks, they rarely become infected. In a review of cases in the United States, where most infections are caused by Sin Nombre virus, 12 (7.5%) of 160 patients were grouped in clusters. The pattern of case manifestations and the fact that the members of each cluster were exposed to sites with large infestations of rodents induced the authors to conclude that these clusters originated from exposure to common rodent sources (*11*).

In our study, 39.2% of the cases were grouped in clusters; in addition, the 1996 outbreak was an extremely large cluster (*4*,*5*). Patients in our cluster A lived in different towns and became sick almost simultaneously, 3 weeks after coming back from a wild area where they shared high-risk activities. These characteristics suggest simultaneous exposure to a rodent source. Unlike the previous category, type B clusters were characterized by an index case-patient, who almost always died, followed 19–40 days later by the illness of >1 more close contacts of that patient.

Although the incubation period for human hantavirus illnesses is 8–45 days, it usually lasts 2–3 weeks (*2*). If the members of each cluster type B were infected after a common exposure, the incubation period of the patients with secondary cases would result from adding the estimated incubation period for the index patient (2–3 weeks) to the intervals between index and secondary cases. Under this hypothesis, the incubation period for secondary cases to develop would be abnormally long and rarely probable. On the other hand, the average interval between index and secondary cases in type B clusters (23.4 days) was similar to the average incubation period accepted for hantavirosis. The former also matched the average interval among 1996 outbreak case-patients (22.8 days) that was associated with the incubation period for person-to-person transmission. Therefore, intervals between cases in type B clusters suggest person-to-person transmission. In these clusters no transmission from secondary case-patients was detected, in contrast with the 1996 outbreak in which up to 4 link chains were identified (*4*,*5*).

Another explanation for the long intervals could be that infected peridomestic rodents contaminate the environment, resulting in multiple transmissions over an extended period. However, this possibility seems unlikely for 4 reasons. First, for clusters B7 and B8, occupational exposures, considered as the most likely risk for the index case-patients, were excluded for the secondary case-patients. In cluster B2, 3 of the patients shared a possible common exposure (fishing excursion), but the fourth (index case-patient’s daughter) did not. Second, immediate actions to eliminate risk sources were taken when a patient was detected, which lowered the risk for persisting domestic or peridomestic rodent sources. Third, evidence of domestic rodent infestation was absent for some clusters. Fourth, no serologic reactivity was detected in rodents captured in peridomestic areas. Overall, although in any other hantavirus outbreaks, multiple virus introductions to humans from the environment are possible, clusters like type B are the exception.

In any case, in the southern region of Argentina, rodent exposure risks are difficult to exclude. Even in urban zones, wild vegetation is intermingled with settlements, and close contact with rural areas allows the circulation of *Oligoryzomys longicaudatus*. This fact delayed the suspicion for interpersonal transmission in the 1996 outbreak because the first case-patients lived in or visited towns with these characteristics. Person-to-person transmission was first strongly suspected when 1 patient was transferred to a hospital outside of the Andes virus–endemic area. The admitting physician, who had no other risk factor for exposure, became ill with HPS 3 weeks later (*3*). Recently, in a reported cluster caused by Andes virus infection, the journey of the index case-patient out of the Andes virus–endemic area was also the key to suspecting person-to-person transmission (*12*,*13*). Therefore, person-to-person transmission is evident only when special circumstances converge, as happens when an infected patient spreads the virus out of the disease-endemic area. In this situation, molecular studies are especially useful because identical sequences in geographically separated but linked cases support interpersonal transmission (*5*,*12*,*13*). However, when all case-patients remain in the disease-endemic area, infection may be attributed to other sources, hiding the interpersonal transmission. In this situation, molecular dissimilarities rule out person-to-person transmission, but identical sequences do not support it. The interval length may be helpful: a 2- to 4-week interval among linked patients supports interpersonal transmission, whereas a shorter interval suggests simultaneous exposure. Reported clusters of Andes virus infection in southern Chile showed the 2 patterns; some cases had intervals of 2–5 days, and others had intervals of >2 weeks between cases (*14*,*15*).

An infection with high levels of virus replication might correlate with the severity of the patient’s illness and result in increased shedding of virus, which would initiate secondary cases. Subsequent spread of the virus through human hosts might induce a reduction of the initial virulence, which would explain the lower death rate and mild forms observed among patients with secondary cases. The 1996 outbreak may be explained by the circulation of a uniquely virulent and transmissible virus strain or an unusually high viral replication in a particular patient. However, the human-to-human spread may show idiosyncratic behavior of Andes virus as well as an extraordinary situation. From this point of view, the 1996 outbreak can be considered the maximum expression of person-to-person spread. Human infections by Andes virus have also shown distinctive clinical characteristics with more hemorrhagic, renal, hepatic, and muscular impairment than those reported for Sin Nombre virus infection (*16*–*20*).

Although the mechanisms of person-to-person transmission are still not clear, direct contact and aerosol transmission must first be considered. Direct contact was always taking place between family members (type B clusters); because cough appears at the end of the febrile phase, saliva may play an important role in transmission during the early stages (as suggested by the detection of virus antigen in rodents’ saliva glands) (*21*). For infected couples, sexual contact should also be considered, and breast-feeding cannot be excluded in mother-baby clusters. Aerosol infectivity can be suspected between persons because the natural spread from rodents to persons is by the aerosol route (*22*). Respiratory secretions may be sources of infection because pulmonary involvement is essential in HPS, viral antigen is present in pulmonary endothelium (*23*,*24*), and Andes virus RNA has been reported in tracheal secretions of infected persons (*25*).

Although in the 1996 outbreak, hospitals played a key role in amplification, nosocomial transmission of Andes virus seemed to be the exception (*4*,*5*). Under usual circumstances, the period of transmission is probably short and limited to the early phase. Close and prolonged contact may be necessary for interpersonal transmission. Generally, the infection has already progressed to the cardiopulmonary phase at the time of patient’s admission, so healthcare workers are exposed during a late stage and do not have close contact with the patient if they take adequate biosafety measures. These facts may explain the absence of HPS cases in healthcare workers during the period reviewed and the low seroprevalence reported in this group, which is similar to that of the general population of the region (*26*,*27*). Seroprevalence in healthcare workers may not be a sensible indicator of the need to investigate person-to-person transmission of Andes virus because such transmission mostly occurs in the patient’s domestic circle during the early stages of the illness. In any situation, universal precautions should be strictly followed. Surgical masks with visor, gowns, and gloves should be routinely worn and, whenever possible, additional measures such as using HEPA respirators and providing private rooms should be used to protect against inspired particles.

Even though virus characterization was not performed for all the cases reviewed, Andes virus is the unique genotype identified since 1995 in infected persons and rodents in the southern region of Argentina. For this reason, Andes virus is the most probable etiologic agent in those cases not characterized.

The number of clusters identified during the period reviewed is high compared with the low incidence of HPS in the region. This finding suggests that person-to-person transmission of Andes virus is not exceptional and must always be suspected when the onset of symptoms of >2 case-patients linked by contact are separated by an interval of ≈3 weeks. Case-patients with an ultimately fatal disease have an increased risk of initiating secondary cases.

Surveillance of household contacts is useful for identifying mild symptoms. Contacts should be encouraged to seeking immediate medical care if febrile symptoms appear, and specific diagnostic tests must be performed.
